# Overexpression of Bcl2 abrogates chemo- and radiotherapy-induced sensitisation of NCI-H460 non-small-cell lung cancer cells to adenovirus-mediated expression of full-length TRAIL

**DOI:** 10.1038/sj.bjc.6601910

**Published:** 2004-06-01

**Authors:** M A I Abou El Hassan, D C J Mastenbroek, W R Gerritsen, G Giaccone, F A E Kruyt

**Affiliations:** 1 Department of Medical Oncology, VU University Medical Centre, Amsterdam, The Netherlands

**Keywords:** NSCLC, full length TRAIL, adenovirus, synergy, Bcl-2

## Abstract

TNF-related apoptosis-inducing ligand (TRAIL, also known as Apo-2L) is a promising novel anticancer agent that selectively induces apoptosis in tumour cells and the activity of which can be enhanced by combined treatment with chemo- or radiotherapy. For therapeutic purposes, the use of full-length TRAIL may be favourable to recombinant TRAIL based on its increased tumour cell killing potential, and the delivery of TRAIL at the tumour site by adenovirus vectors may provide an approach to overcome the short half-life of recombinant TRAIL and hepatocyte toxicity *in vivo*. Here, we constructed an adenoviral vector expressing full-length TRAIL (AdTRAIL) and studied the potential of chemo- and radiotherapy in enhancing AdTRAIL-induced apoptosis in non-small cell lung cancer (NSCLC) H460 cells and normal cells and, in addition, investigated the mechanism of AdTRAIL-induced apoptosis. AdTRAIL effectively killed H460 cells, which we previously showed to have a deficiency in mitochondria-dependent apoptosis by downstream activation of caspase-8 rather than caspase-9. Further analyses revealed that AdTRAIL induces death receptor- and mitochondria-dependent apoptosis that could be partially suppressed by Bcl2 overexpression. Combined treatment with doxorubicin (DOX), cisplatin (CDDP), paclitaxel (PTX) and radiation strongly enhanced AdTRAIL-induced cytotoxicity in a synergistic way. Synergy was accompanied by the cleavage of Bid and an increase in caspase-8 processing that was abolished by Bcl2 overexpression, indicating that the Bid-mitochondrial amplification loop is functional in H460 cells. Moreover, combination treatment did not alter the tumour selectivity of AdTRAIL since normal human fibroblasts (NHFs) remained resistant under these conditions. These findings further indicate that the combined use of chemo/radiotherapy and adenovirus-produced full-length TRAIL may provide a valuable treatment option for NSCLC.

The treatment of advanced cancer, including non-small-cell lung cancer (NSCLC), is often hampered by the intrinsic or developing resistance against the anticancer drugs used (De Vita *et al*, 2001). In this context, deregulated apoptosis in cancer can contribute to drug resistance since the apoptosis-inducing ability of therapeutic agents is at least partially responsible for drug efficacy. Efforts to circumvent drug resistance include the combined use of different drugs with different mechanisms of action to enhance the overall antitumour effect, which for example can be based on the activation of distinct or overlapping apoptotic pathways that in a cooperative manner can trigger apoptosis more effectively.

TNF-related apoptosis-inducing ligand (TRAIL, also known as Apo-2L) represents a novel promising anticancer agent whose activity is solely dependent on its ability to induce apoptosis in tumour cells (Ferreira *et al*, 2002; MacFarlane, 2003). Unlike other members of the TNF super family (TNF and FasL), TRAIL acts as a specific antitumour agent without harming normal cells (Huang *et al*, 2002; Lin *et al*, 2002a, 2002b). TNF-related apoptosis-inducing ligand is a type-II transmembrane protein and shows the highest homology to FasL. The extracellular domain of TRAIL forms a soluble molecule upon cleavage (Wiley *et al*, 1995; Pitti *et al*, 1996). Both soluble and full-length TRAIL bind to their cognate cell surface receptors in the target cell to engage the apoptotic pathway (Ashkinazi *et al*, 1999) although the two TRAIL variants have been reported to possess different apoptosis-inducing capacities in cancer cells suggesting currently unresolved differences in their mechanism of action (Voelkel-Johnson *et al*, 2002; Seol *et al*, 2003). Of the five TRAIL receptors identified so far, TRAIL-R1 (DR4), TRAIL-R2 (DR5/TRICK/KILLER) and TRAIL-R4 (DcR1/TRUNDD) encode classical type-I transmembrane proteins (Pan *et al*, 1997a, 1997b). The TRAIL-R1 and -R2, which signal for apoptosis, have a complete cytoplasmic death domain (DD) while R3 and R4 act as decoy receptors having either no or a truncated cytoplasmic domain, respectively.

In animal experiments, TRAIL did not cause systemic toxicity (Kagawa *et al*, 2001). It was however reported that recombinant his-tagged TRAIL could cause toxicity to human, but not murine - or non-human primate hepatocytes *in vitro* (Jo *et al*, 2000), which has been assigned to conformational changes in TRAIL structure caused by the histidine tag (Lawrence *et al*, 2001). Apart from the issue of hepatotoxicity, soluble TRAIL has demonstrated a potent antitumour activity against a wide range of tumours both *in vitro* and *in vivo* (Pitti *et al*, 1996; Nimmanapalli *et al*, 2001; Rohn *et al*, 2001; Naka *et al*, 2002).

The applicability of soluble TRAIL in cancer therapy, however, is limited by its short half-life *in vivo* (Kelley *et al*, 2001) that may be overcome by the production of TRAIL at the tumour site by for example a nonreplicating adenoviral vector, which will also reduce the risk of hepatotoxicity (see also Griffith *et al*, 2000; Lin *et al*, 2002a).

The mechanism of TRAIL-induced apoptosis has been studied and the majority of cells can be classified as type I, that is, TRAIL-induced apoptosis is solely mediated by the death receptor pathway (Walczak *et al*, 2000). In this case, the binding of TRAIL to its death receptors triggers the aggregation of the death-inducing signal complex (DISC) with subsequent caspase-8 activation and the activation of executioner caspases and consequently irreversible apoptosis. On the other hand, type-II cells, including several colon carcinoma, neuroblastoma and NSCLC cell lines, are characterized by a strong involvement of the mitochondrial pathway via the caspase-8-dependent activation of the proapoptotic Bcl2 family member Bid, also known as the amplification loop (Sun *et al*, 2001; Fulda *et al*, 2002; Ozoren and El-Deiry, 2002). In addition, the combined treatment with different chemotherapeutic agents or ionising radiation is known to enhance the antitumour activity of soluble TRAIL in additive or synergistic manners, both *in vitro* and *in vivo* models, and has been related to the increased activation of the mitochondrial pathway but also to the enhanced expression of TRAIL receptors (Gibson *et al*, 2000; Held and Schulze-Osthoff, 2001; Mitsiades *et al*, 2001; Xu *et al*, 2003).

In this study, we examined the apoptosis-inducing effect of a constructed adenoviral vector expressing full-length TRAIL in the NSCLC cell line NCI-H460 and in normal cells when applied alone or in combination with different types of chemotherapeutic agents and radiation. It should be noted that NSCLC cells have a deficiency in the mitochondrial apoptotic pathway in that caspase-8 is activated rather than caspase-9 caused by a yet unknown disturbance in apoptosome functioning (Ferreira *et al*, 2000). Owing to this and the relative lack of knowledge on the mechanism underlying full-length TRAIL-induced apoptosis, we also addressed the functioning of the mitochondria amplification loop in this context.

Our findings indicate that adenoviral expression of full-length TRAIL in combination with chemo/radiotherapy may provide an effective and selective strategy for the treatment of NSCLC.

## MATERIALS AND METHODS

### Cell culture and treatment

Human non-small-cell lung adenocarcinoma (NSCLC) NCI-H460 cells and Bcl2 stable overexpressing derivatives (H460Bcl2, described earlier (Ferreira *et al*, 2000) were cultured in RPMI 1640 medium (Invitrogen, Breda, The Netherlands) and normal human fibroblasts (NHF) in Nutrient mixture F10 (Invitrogen). Both media were supplemented with 10% heat-inactivated foetal calf serum (Invitrogen, Breda, The Netherlands), 50 IU ml^−1^ penicillin, 50 *μ*g streptomycin and 1 *μ*g ml^−1^ puromycin (only for H460Bcl2 cells) and cells were grown at 37°C in a humidified air with 5% CO_2_. Cell lines were routinely tested for the absence of mycoplasma infection before use. The expression of Bcl2 protein was confirmed by immunohistochemistry before starting the experiments (data not shown). For optimal adenoviral infection, near-confluent cell cultures were used throughout the study. Cells were treated with doxorubicin (DOX, purchased from Pharmacia Upjohn BV (Woerden, The Netherlands), paclitaxel (PTX, purchased from Sigma, Zwijndrecht, The Netherlands), cisplatin (CDDP, purchased from Pharma Chemie BV, Harlem, The Netherlands) or 6 Gy ionising radiation (80-kV orthovolt X-ray source (Pantak Therapax SXT 150)). For caspase inhibition, the synthetic inhibitor zVAD-fmk (Enzyme System Products, Livermore, CA, USA) was used.

### Adenovirus construction

The TRAIL open reading frame (ORF), kindly provided by Dr H Yagita, Juntendo University School of Medicine, Japan, was cloned under the control of the immediate early cytomegalovirus (CMV) promoter in the pShuttle plasmid and subsequently recombined with the pAdeasy plasmid for the production of replication incompetent AdTRAIL The adenovirus vectors were propagated in 293 cells and purified by CsCl density gradient. The viral preparations were dialysed and stored at −80°C until use.

The titer of AdTRAIL stock, as determined by the limiting dilution assay, was 3.5 × 10^9^ plaque forming unit (pfu)/ml and the viral particle to pfu ratio measured at OD_260_ was less than 60. The adenovirus stocks were free of replication competent adenovirus (RCA) as tested by PCR using primers flanking the AdE1A region (Abou El Hassan *et al*, 2003). The adenovirus expressing green fluorescence protein (GFP) was used as a control and was described earlier (Van Beusechem *et al*, 2000).

### Infection and cytotoxicity measurement

H460, H460Bcl2 and NHF cultured in 96-well plates were incubated with different multiplicity of infection, that is, virus to cell ratio (MOI) of AdTRAIL as indicated in growth medium (50 *μ*l/well) at 37°C. At 2 h postinfection, another volume of virus-free growth medium was added. Cytotoxicity of AdTRAIL alone or combined with other treatments was determined by MTT assays as described previously (Abou El Hassan *et al*, 2003a).

The percentage survival (taking the blank as 100% survival) was plotted as a function of MOI or drugs concentration. The LC_50_ values of each drug with (out) preinfection with AdTRAIL – that is, the concentration of drug required to kill 50% of the cultured cells – were determined and the derived sensitisation factors were calculated as the ratio of the LC_50_ of drug alone/LC_50_ of drug with AdTRAIL infection.

### Western blotting

Treated cells were lysed 24, 48 and 72 h postinfection with RIPA buffer (50 mM Tris-HCl, 150 mM NaCl, 0.1%. SDS, 0.5% sodium deoxycholate (DOC, Fluka Biochemika, Buchs, Switzerland) and 1% nonidet P40 (NP40, Fluka Biochemika)). The cellular lysates were immediately stored at −80°C. Protein (50 *μ*g) of each sample (determined by Biorad total protein assay (Bio-Rad Laboratories BV, Veenendaal, The Netherlands) were separated on a 12.5% SDS–PAGE. Thereafter, proteins were blotted on Immobilon membrane (Millipore BV, Etten-Leur, The Netherlands) and subsequently incubated in blocking solution containing 5% nonfat milk in TBST (0.2% Tween 20, 150 mM NaCl and 10 mM Tris-HCl, pH 8).

The following primary antibodies were used: rabbit polyclonal anti-human TRAIL (dilution 1 : 1000; PeproTechLTD, London, UK), rabbit polyclonal anti PARP (dilution 1 : 2000; Roche, Basel, Switzerland), anti-caspase 8 mAb (dilution 1 : 2000; Immunotech, Prague, Czech Rep), rabbit polyclonal anti-Bid (dilution 1 : 2000; Roche) and anti-*β*-actin mAb (dilution 1 : 7500; Sigma, St Louis, MO, USA). After incubation for 1–2 h with the primary antibody and washing in TBST, the blots were incubated with peroxidase-conjugated goat anti-rabbit/rabbit anti-mouse 2^ry^ antibody (DAKO, Glostrup, Denmark) (1 : 1250 dilution). For chemoluminescence detection, blots were immersed in Lumi-light plus mix (Roche) and exposed to hyperfilm (Amersham Pharmacia UK Ltd., Bukinghamshire, UK).

### Immunohistochemistry

H460 and NHF cells cultured in 96-well plate were infected with AdTRAIL (MOI 100). At 3 days postinfection, cells were fixed with chilled methanol : acetone mix (1 : 1, v v^−1^). TNF-related apoptosis-inducing ligand expression was monitored with rabbit polyclonal anti-human TRAIL antibody (dilution 1 : 200; PeproTechLTD), for 1 h at 37°C. After washing with PBS, cells were incubated with peroxidase-conjugated goat anti-rabbit secondary antibody. The stained cells were visualised using AEC substrate chromogen (DAKO).

## RESULTS

### Construction and characterisation of AdTRAIL

**Figure 1:**
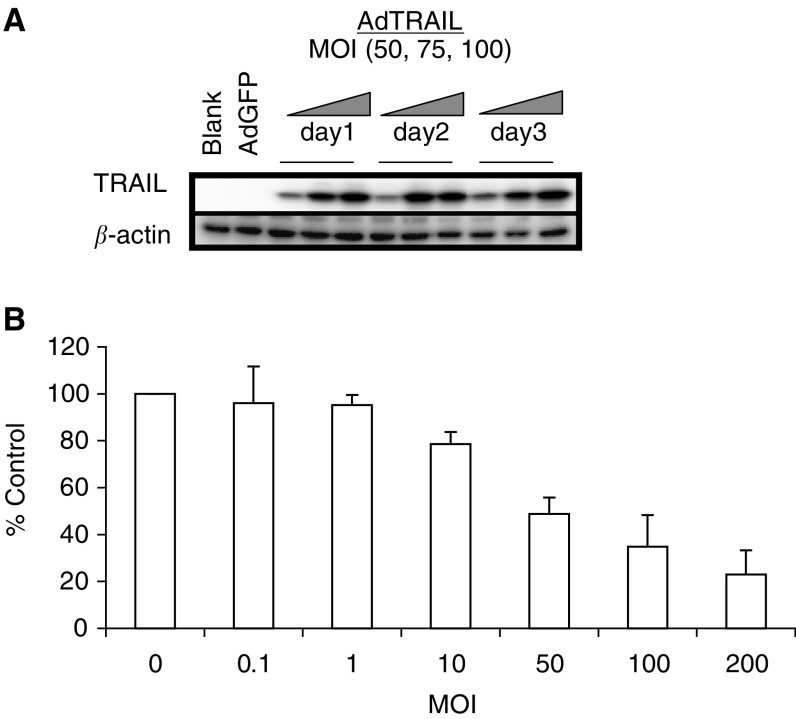
TNF-related apoptosis-inducing ligand expression and survival of Ad-TRAIL-infected H460 cells. Cells were infected with different MOIs of AdTRAIL, and TRAIL expression was determined by Western blotting at 1, 2 and 3 days postinfection (**A**). Survival of infected cells was determined by MTT assays at 3 days postinfection (**B**).

An adenoviral vector expressing full-length TRAIL was constructed as described in the Materials and Methods section. Figure 1A

shows the expression of TRAIL in H460 cells followed up to 3 days postinfection with different MOIs of AdTRAIL. The cellular expression of TRAIL was MOI dependent and reached its maximal level at 1 day postinfection. Further increases in TRAIL expression were likely limited by the toxic effect of TRAIL on the infected producer cells. The MOI-dependent killing of H460 cells by AdTRAIL was confirmed 3 days postinfection by MTT assays as shown in Figure 1B.

**Figure 2:**
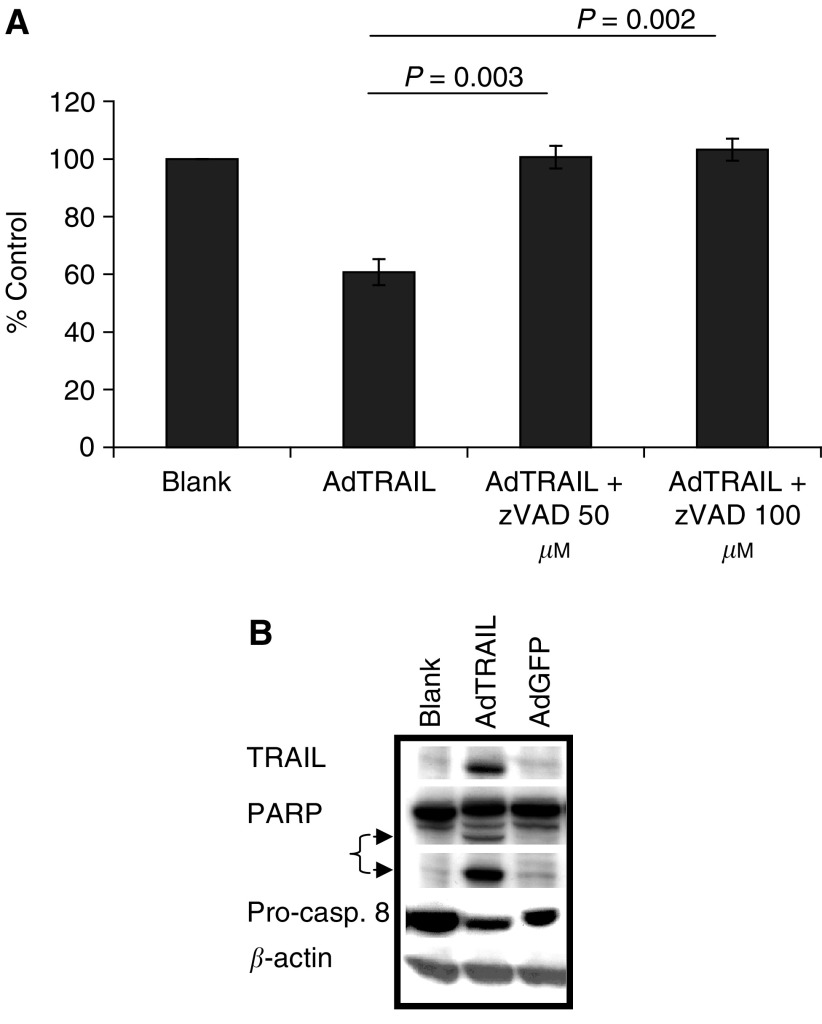
AdTRAIL induces caspase-dependent apoptosis in H460 cells. H460 cells were treated with AdTRAIL (MOI 10) in the presence or absence of zVAD-fmk, and after 3 days postinfection cell viability was determined (**A**). Values are the mean (*n*=3)±s.d., and *P*-values were determined by the Student's *t*-test. Western blot indicating the cleavage of procaspase-8 and PARP (**B**). Arrows indicate the cleaved products of PARP.

To demonstrate apoptosis activation triggered by adenovirus-produced TRAIL, we tested whether the broad caspase inhibitor zVAD-fmk would protect against AdTRAIL toxicity. A complete abrogation of AdTRAIL-induced apoptosis was observed by cotreating H460 cells with different concentrations of zVAD-fmk at 3 days posttreatment (Figure 2A

), indicating the sole involvement of caspases in mediating AdTRAIL-induced apoptosis. To further characterise caspase dependency of AdTRAIL-induced apoptosis, the cleavage of procaspase-8 and PARP was determined in H460 cells 3 days postinfection with AdTRAIL by Western blotting. AdTRAIL specifically induced procaspase-8 and PARP cleavage as indicated in Figure 2B, when compared to uninfected or AdGFP-infected cells.

### Combination treatment of AdTRAIL with chemo(radio)therapy

Figure 3

**Figure 3:**
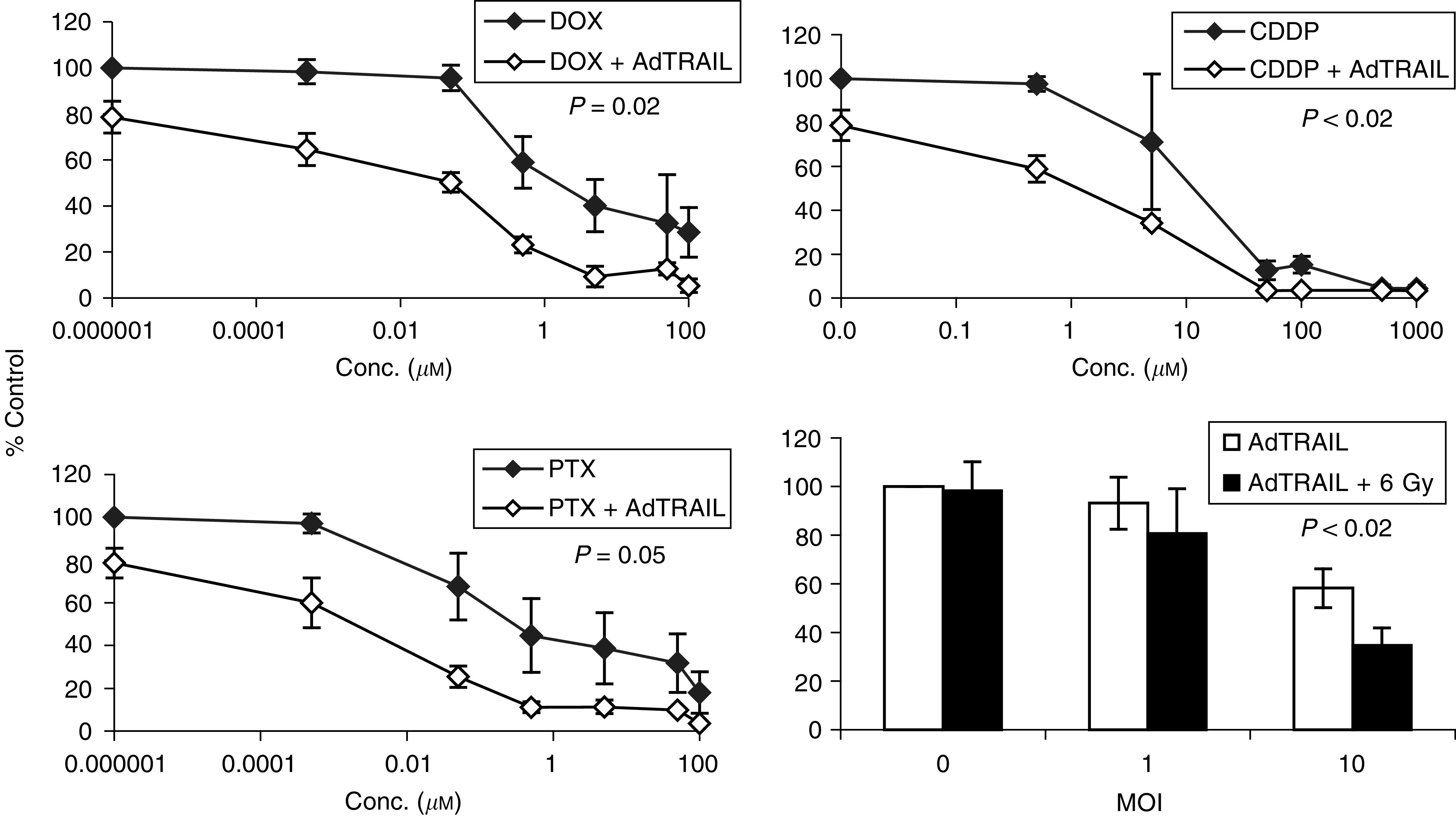
Cytotoxic effect of AdTRAIL (MOI 10) alone or in combination with different concentrations of DOX, PTX, CDDP or 6 Gy irradiation in H460 cells at 3 days postinfection. Values are the mean (*n*=3)±s.d. The *P*-values were calculated by comparing the slopes of the multiple regression of ln (viability2) of H460 cells treated with AdTRAIL alone or combined with DOX, PTX or CDDP *vs* ln (drug conc.^2^) by Student's *t*-test. For the irradiated groups, the *P*-value was calculated by comparing the viability of cells treated with AdTRAIL alone or in combination with 6 Gy irradiation using Student's test.

shows the combined effect of different types of chemotherapeutic drugs, DOX, PTX, CDDP or 6 Gy irradiation with AdTRAIL (MOI 10) on the viability of H460 cells at 3 days after treatment. Under these conditions, AdTRAIL caused moderate cell killing in H460 cells with a viability of 78.6±6.9% of control noninfected cells. Doxorubicin, PTX or CDDP alone induced a concentration-dependent cell kill of H460 cells with LC_50_ values of 1.5, 0.3 and 15 *μ*M, respectively (see also Table 1

**Table 1:** AdTRAIL-induced sensitisation of H460, H460Bcl2 and NHF cells to DOX, PTX or CDDP 2 days after treatment

	**LC_50_ (mM)**					
	**DOX**		**PTX**		**CDDP**	
	−	**+AdTRAIL**	−	**+AdTRAIL**	−	**+AdTRAIL**
H460	1.5	0.015	0.3	0.002	15	1.5
SF^a^		100		150		10
H460Bcl2	0.5	0.25	0.1	0.005	15	15
SF^a^		2		20		1
NHF	3	3	90	85	28	35
SF^a^		1		1.1		0.8

a Sensitisation factor.

DOX=doxorubicin; CDDP=cisplatin; PTX=paclitaxel; LC_50_=the concentration of drug required to kill 50% of the cultured cells; NHFs=normal human fibroblasts.

). Interestingly, the combined treatment with subtoxic concentrations of DOX, PTX or CDDP already sensitised H460 cells to AdTRAIL-induced apoptosis (*P*⩽0.05, Figure 3). Accordingly, the LC_50_ values of DOX, PTX and CDDP were reduced 100-, 150- and 10-fold, respectively (Table 1). Likewise, the use of 6 Gy ionising radiation, which did not result in cytotoxicity in H460 cells, augmented the apoptotic effect of AdTRAIL in H460 cells at 3 days postinfection.

**Figure 4:**
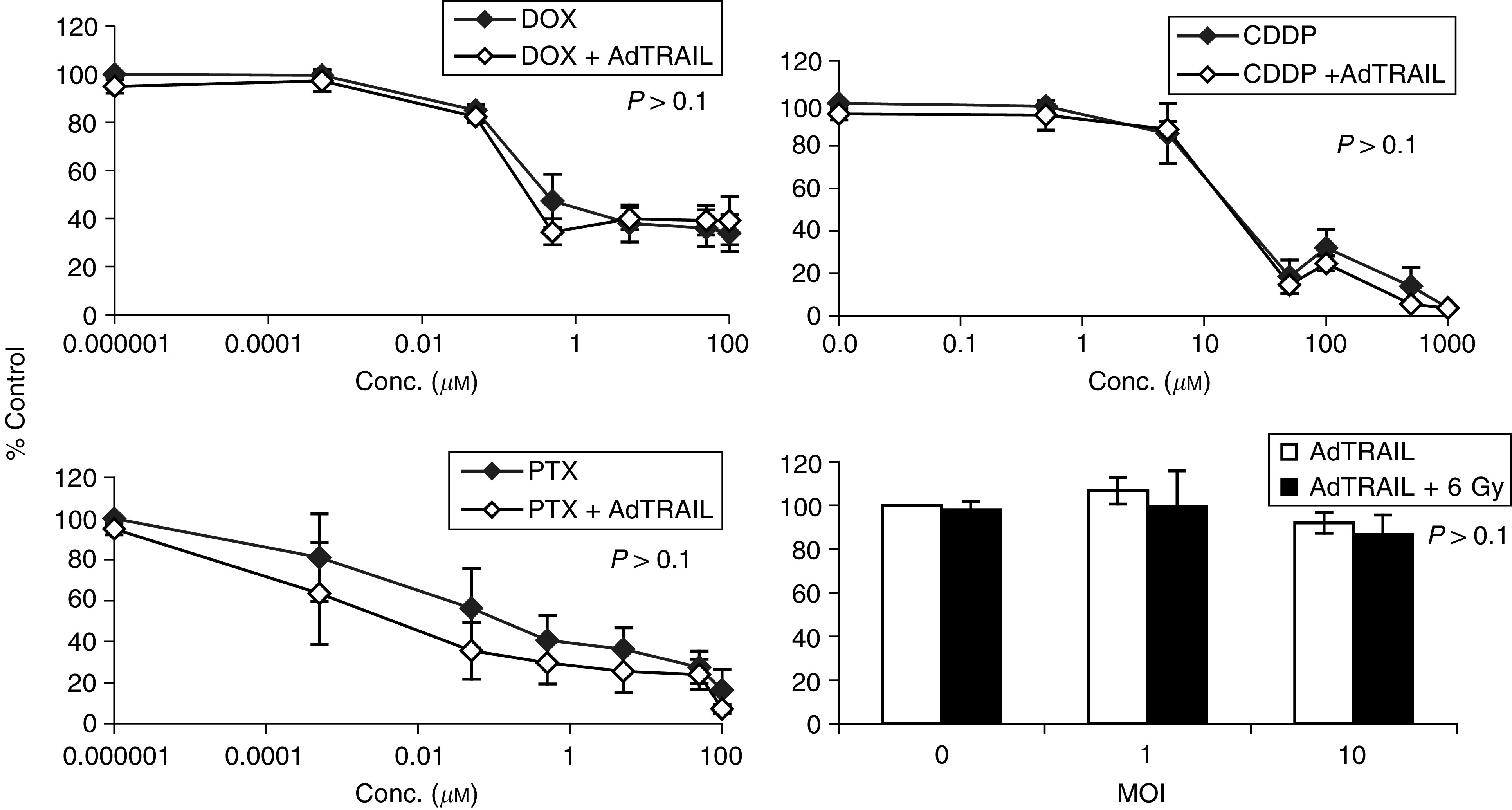
Cytotoxic effect of AdTRAIL (MOI 10) alone or in combination with different concentrations of DOX, PTX, CDDP OR 6 Gy irradiation in H460Bcl2 cells at 3 days postinfection. Values are the mean (*n*=3)±s.d. The *P-*value was calculated by comparing the slopes of the multiple regression of ln (viability2) of H460Bcl2 cells treated with AdTRAIL alone or combined with DOX, PTX or CDDP *vs* ln (drug conc.2) by Student's *t* test. For the irradiated groups, the *P-*value was calculated by comparing the viability of cells treated with AdTRAIL alone or in combination with 6 Gy irradiation using Student's test.

In order to study whether the mitochondria amplification loop mediates the observed synergistic effects in the mitochondria/caspase-9 pathway impaired H460 cells, Bcl2-overexpressing cells (H460Bcl2) were treated with AdTRAIL alone or combined with chemotherapy or irradiation. The overexpression of Bcl2 significantly protected H460 cells against AdTRAIL-induced apoptosis (compare Figures 3 and 4

).

In addition, H460Bcl2 cells could not be sensitised to AdTRAIL by DOX, CDDP and 6 Gy-irradiation, whereas the sensitising effect of PTX was strongly reduced but not completely abolished (*P*>0.1, Figure 4 and Table 1). As control, the combined treatment with chemo (radio)therapy did not sensitise H460 cells infected with the AdGFP (MOI 100) control virus (data not shown).

### Bcl2 overexpression prevents caspase-8 activation and Bid cleavage

**Figure 5:**
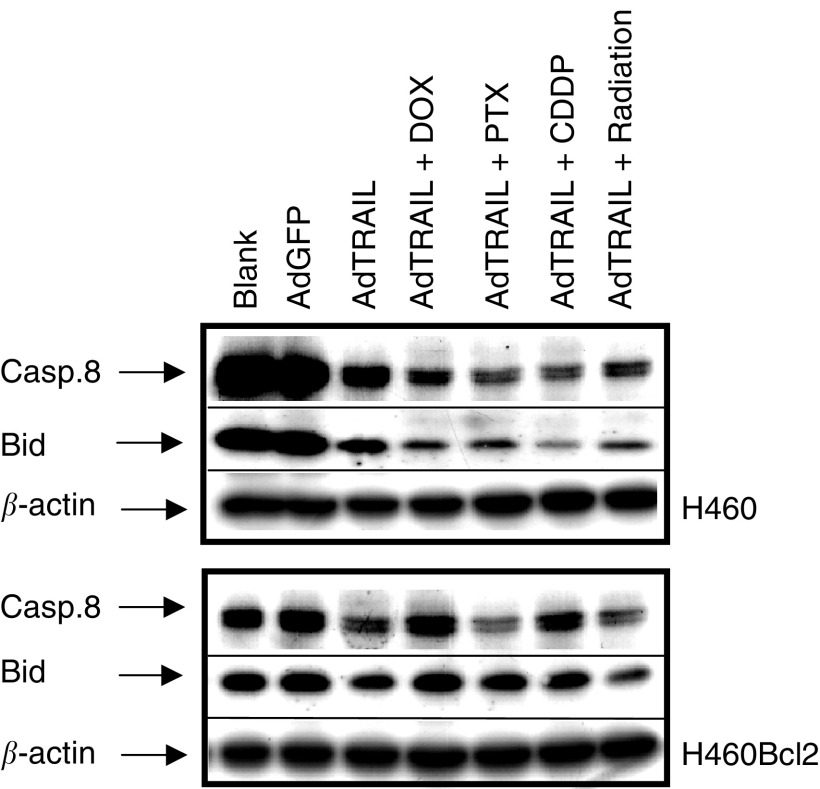
Bid and procaspase-8 cleavage in H460 and H460Bcl2 cells 3 days postinfection with AdTRAIL (MOI 10) with (out) 15 nM of DOX or PTX, 15 *μ*M CDDP or 6 Gy irradiation.

To further examine the role of Bcl2 in preventing the potentiating effect of chemotherapy and ionising radiation to AdTRAIL-induced apoptosis, we studied the cleavage of procaspase-8 and Bid in H460 and H460Bcl2 cells after infection with AdTRAIL (MOI 10) alone or in combination with 15 nM DOX or PTX, 15 *μ*M CDDP or 6 Gy irradiation 3 days postinfection. Figure 5

shows that control adenovirus infection (AdGFP) did not induce caspase-8 activation or Bid cleavage in H460 and H460Bcl2 cells as indicated by the constant levels of the nonprocessed forms. AdTRAIL infection induced caspase-8 and Bid cleavage, which was enhanced upon combined treatment with chemo (radio) therapy in H460 cells. The overexpression of Bcl2 almost completely prevented the cleavage of Bid in cells treated with AdTRAIL alone and when combined with chemo (radio) therapy, and also reduced procaspase-8 cleavage. This indicates that the lack of chemo/radiation-dependent sensitisation of H460Bcl2 cells to AdTRAIL is related to the Bcl2-dependent prevention of the enhanced processing of procaspase-8 and Bid.

### Effect of AdTRAIL combination treatment on normal cells

**Figure 6:**
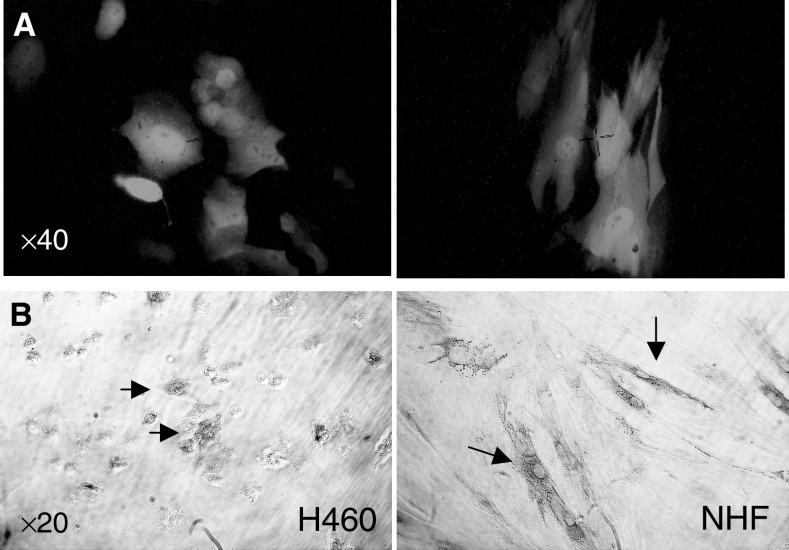
AdGFP and AdTRAIL infection of H460 and NHF cells. Cells were infected at MOI 100, and after 3 days, GFP expression (**A**) or TRAIL expression (**B**) was determined.

To study the possibility that under conditions of chemotherapy enhanced Ad TRAIL-induced H460 cell killing the sensitivity to normal cells could also be altered, we treated NHF cells in the same way and determined cytotoxicity. For this purpose, first the infection efficiency of NHF was examined. Figure 6A

shows a considerable level of AdGFP infection at MOI 100 in NHF cells of approximately 20%, whereas H460 cells demonstrated an infection efficiency of around 70%.

**Figure 7:**
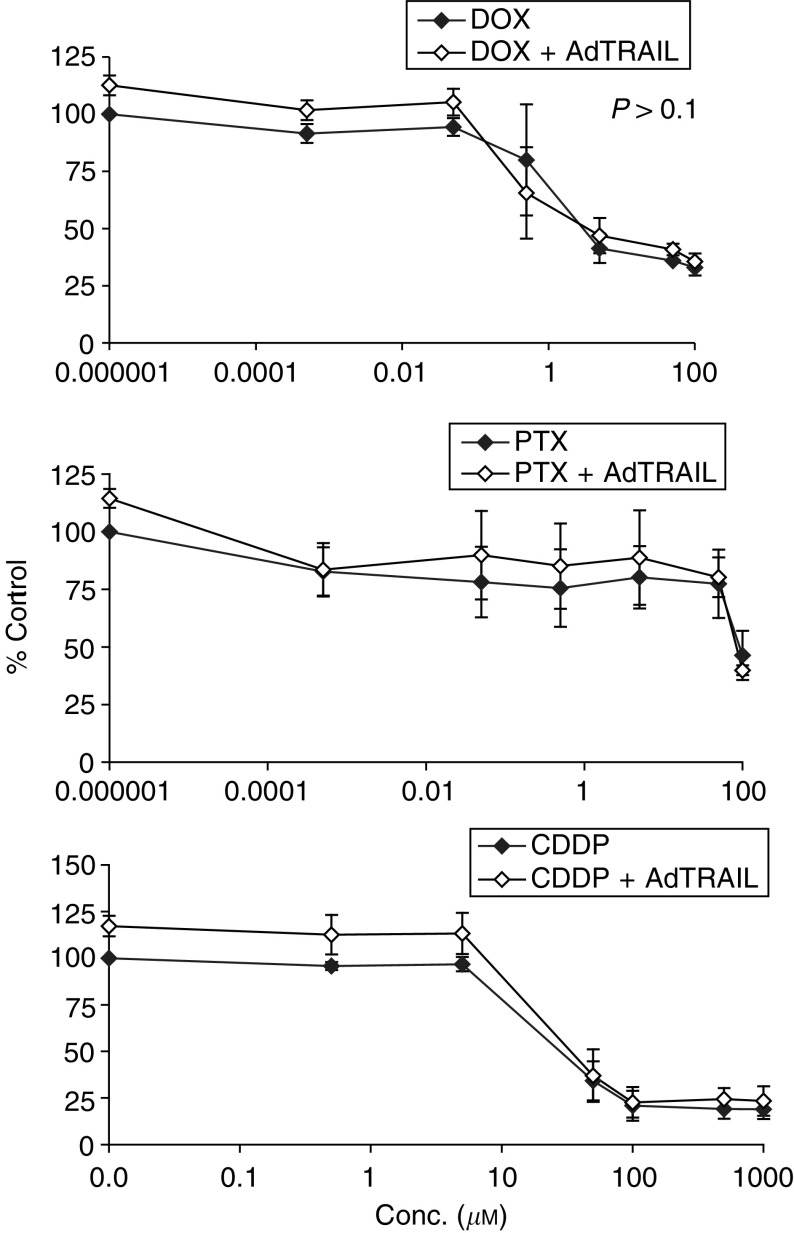
Chemotherapy in combination with AdTRAIL does not alter the sensitivity of NHF. The cytotoxic effect of AdTRAIL (MOI 100) with (out) different concentrations of DOX, PTX and CDDP was determined at 3 days postinfection. Values are the mean (*n*=3)±s.d. The *P*-value was calculated by comparing the slopes of the multiple regression of NHF cells ln (viability2) treated with AdTRAIL alone or combined with DOX, PTX or CDDDP *vs.* ln (drug conc.^2^) by Student's *t*-test.

Infection with AdTRAIL eradicated most of the H460 cells but left the NHF intact as indicated by the few remaining cells that were either positive or negative for TRAIL expression in H460 cells as determined by immunohistochemistry (Figure 6B). Subsequently, the effect of combined chemotherapy at different concentrations with AdTRAIL on NHF cells was examined (Figure 7

). Each of the drugs alone exerted a concentration-dependent cytotoxicity on NHF cells 2 days post-treatment. Normal human fibroblasts were relatively refractory to the damage induced by the chemotherapeutic agents (especially to PTX) when compared to H460 cells (see Table 1). The cotreatment of NHF with different concentrations of DOX, PTX or CDDP 24 h postinfection with AdTRAIL did not result in extra cytotoxicity either compared to AdTRAIL alone or chemotherapy alone. Instead, perhaps even an increase in viability after AdTRAIL infection could be appreciated (Figure 7). These data show that adenovirus-expressed TRAIL combined with chemotherapy does not alter the sensitivity of normal cells.

## DISCUSSION

Adenovirus-mediated production of the promising biological anticancer agent TRAIL is a form of proapoptotic gene therapy that has gained considerable attention recently (Griffith *et al*, 2000; Routes *et al*, 2000; Lin *et al*, 2002b). The main reasons for this are the intrinsic tumour-selective activity of TRAIL in a broad range of cancer cells that complements the rather nonselective delivery of viral vectors to tumours and normal tissues, the observed bystander effect of TRAIL resulting in the killing of cells surrounding the infected cells (Kagawa *et al*, 2001), and the notion that virally produced TRAIL may overcome problems observed with the use of recombinant soluble TRAIL regarding protein instability and resistance (Kelley *et al*, 2001; Voelkel-Johnson *et al*, 2002; Seol *et al*, 2003).

Full-length TRAIL has recently been reported to kill tumour cells that are resistant to soluble TRAIL, suggesting a greater therapeutic potential of this form and indicating the existence of yet not understood mechanistic differences between the two TRAIL variants (Voelkel-Johnson *et al*, 2002; Seol *et al*, 2003).

The tumour-selective properties of full-length TRAIL alone or in combination with chemo- and radiation treatment thus far have been mainly investigated and confirmed for recombinant TRAIL, and its mechanism of action has been left unexplored.

In the present study, we generated an adenoviral vector expressing full-length TRAIL to explore its possible use for the treatment of NSCLC. We used the NSCLC cell line H460 as a representative cell line that has been well characterised and in which we found a defect in mitochondria-dependent caspase-9 activation upon treatment with DNA-damaging chemotherapeutic agents that may provide an explanation for chemoresistance of NSCLC in the clinic (Ferreira *et al*, 2000).

AdTRAIL efficiently killed H460 cells in a MOI-dependent manner, which could be inhibited by the broad-caspase-inhibitor zVAD-fmk as an indication of the activation of caspase-dependent apoptosis. AdTRAIL-induced apoptosis in H460 was associated with caspase-8 activation and PARP cleavage. Subtoxic doses of DOX, PTX, CDDP or ionising radiation sensitised H460 cells to AdTRAIL-induced apoptosis that we found to be a result of augmented processing of procaspase-8 and Bid, indicating that both TRAIL and chemo (radio) therapy-induced apoptotic signals converge at the mitochondrial apoptotic pathway. The most likely explanation for this is that both chemotherapeutic agents and irradiation trigger damage-induced signals that lead to the activation of proapoptotic Bcl2 family members, such as Bax and Bak (Cartron *et al*, 2003; Kim *et al*, 2003), and subsequent mitochondria destabilisation that as we reported previously results in the unusual activation of caspase-8 in NSCLC H460 cells by an as yet unresolved mitochondria-dependent mechanism (Ferreira *et al*, 2000). Consequently, Bid cleavage is enhanced and as a result the tBid-dependent amplification loop.

Confirmation for this notion is provided by our finding that the stable overexpression of Bcl2 in H460 cells completely counteracted the enhancing effect of chemo (radio) therapy, except for PTX. Paclitaxel-induced toxicity was significantly reduced by Bcl2 overexpression, but not completely abrogated as found for the other treatments. This may be related to our previous finding that PTX in contrast to, for example CDDP, induces cell death in H460 cells that is only partially triggered through the mitochondrial pathway and mainly depends on the activation of a caspase-independent pathway (Huisman *et al*, 2002). The found importance of the tBid-mitochondria amplification loop in mediating AdTRAIL-induced apoptosis in H460 cells classifies them as type-II cells. This observation is in line with a previous report by Sun *et al* (2001), who showed that Bcl2 overexpression inhibits soluble TRAIL-induced apoptosis in NSCLC cells; however, instead of their suggestion that Bcl2 acts by preventing caspase-7 activation, we find that Bcl2 suppresses procaspase-8 cleavage that as mentioned above we have found to occur in a mitochondria-controlled manner in NSCLC cells (Ferreira *et al*, 2000).

The notion was also tested whether the use of the full-length TRAIL-encoding virus together with chemotherapy may affect the tumour selectivity of the treatment and result in toxic side effects on normal tissue. We did not observe such a change in cytotoxicity in NHF and rather observed a not understood small increase in viability after AdTRAIL infection.

In conclusion, we showed that the production of full-length TRAIL by an adenoviral vector effectively kills NSCLC H460 cells that can be enhanced by combined treatment with the chemotherapeutic agents CDDP, DOX and PTX and by radiation. The synergistic effects are dependent on the enhanced activation of the mitochondria apoptotic pathway that remains functional in the mitochondria/caspase-9 route-deficient H460 cells. Combination treatment of AdTRAIL and chemo/radiotherapy was not toxic for normal cells indicating that adenovirus-directed expression of full-length TRAIL might provide an attractive strategy for treating NSCLC.
